# Facile Fabrication of a Highly Sensitive and Robust Flexible Pressure Sensor with Batten Microstructures

**DOI:** 10.3390/mi13081164

**Published:** 2022-07-23

**Authors:** Xuefeng Zhang, Sheng Chang, Zhixue Tong

**Affiliations:** School of Mechanical and Electrical Engineering, Xi’an University of Architecture and Technology, Xi’an 710055, China; cs@xauat.edu.cn (S.C.); tongzhixue@xauat.edu.cn (Z.T.)

**Keywords:** flexible pressure sensor, surface microstructures, finite element analysis, carbon nanotubes, contact resistance

## Abstract

As the foremost component of wearable devices, flexible pressure sensors require high sensitivity, wide operating ranges, and great stability. In this paper, a pressure sensor comprising a regular batten microstructure active layer is presented. First, the influences of the dimensional parameters of the microstructures on the performances of the sensors were investigated by the mechanical finite element method (FEM). Then, parameters were optimized and determined based on the results of this investigation. Next, active layers were prepared by molding multiwalled carbon nanotube/polyurethane (MWCNT/PU) conductive composite using a printed circuit board template. Finally, a resistive flexible pressure sensor was fabricated by combining an active layer and an interdigital electrode. With advantages in terms of the structure and materials, the sensor exhibited a sensitivity of up to 46.66 kPa^−1^ in the range of 0–1.5 kPa and up to 6.67 kPa^−1^ in the range of 1.5–7.5 kPa. The results of the experiments show that the designed flexible pressure sensor can accurately measure small pressures and realize real-time human physiological monitoring. Furthermore, the preparation method has the advantages of a low cost, simple design, and high consistency. Thus, it has potential to promote the development of flexible sensors, wearable devices, and other related devices.

## 1. Introduction

Wearable sensors can monitor the status of users without interrupting and restricting their actions, so they are of great significance for various potential applications in the future [1,2,3,4,5], including real-time physiological information collection [6,7,8,9], human–machine interaction interfaces [10,11,12], and electronic skin [13,14,15]. In addition, these various practical applications propose various requirements for sensors. For example, pulse and respiration occur in a small pressure range, and the sensor is required to show a higher sensitivity in a low pressure range. However, in the face of human motion, such as wrist movement, plantar pressure measurement, and other activities that output high pressures, sensors need to have a wider measurement range.

It has been proven that the introduction of microstructures in flexible pressure sensors can effectively improve the sensing performance. This is mainly because the microstructure changes the contact pattern of the sensor, reduces the initial contact area, and increases the extent of deformation. In addition, stress concentration is introduced by the microstructure, too. Common microstructures are pyramids [16,17,18,19,20] domes [21,22], and triangular cones. Sun [23] et al. proposed a flexible tactile sensor with a pyramid shape, which could achieve a high sensitivity of 3.2 kPa^−1^ within the range of <1 kPa. Park et al. [24] found that the micro-dome structure showed the best sensitivity to normal, tensile, and bending stresses, and it showed excellent sensitivity over a wide range of pressures. Huang et al. [25] designed a multilayer wave structure piezoresistive sensor based on carbon nanotubes/carbon black/silicone rubber conductive composites, which showed favorable resistance changes in the range of 0–1800 kPa. Chen et al. [26] stamped a Polyvinylidene fluoride copolymer (P(VDF-TrFE)) film with a micropillar array and sandwiched it between a pair of cross-electrode arrays to construct a multichannel multiplexing sensor array, which could be applied to dynamic tactile monitoring. In addition, Zhang et al. [27] used laser sintering (SLS) technology to produce an irregular microstructure on the powder surface, allowing the sensor to have a wide linear range of 0–100 kPa. Andreia dos Santos et al. [28] proposed a new method for the microstructure of a PDMS film fabricated with a laser engraving mold, which created a vertebral body structure on the surface and allowed the sensor sensitivity to reach 2.5 kPa^−1^. Zhu et al. [29] treated a micro-pyramid silicon template with a combination of photolithography and wet etching to form anisotropic microstructure arrays, which were assembled with graphene into ultrahigh-performance (sensitivity of 5.53 kPa^−1^ and good stability) resistive tactile sensors. The above studies show that the microstructures make sensors more sensitive under pressure.

Even the introduction of microstructured films has a positive effect on the sensitivity of flexible devices. However, the fabrication of these microstructures usually requires lithography or laser-formed templates, which is complex, expensive, and time-consuming. In this sense, flexible pressure sensors involved with microstructures achieved by lithography have hindered further applications of such devices. Therefore, researchers have proposed a method of employing natural biomaterial templates, which is economic and easily performed. This method is mainly based on a natural surface that possesses a particular and superior form with microstructures that are appropriate for flexible pressure sensors. For example, Yu et al. [30] reported a novel photo-enhanced high-sensitivity piezoresistive pressure sensor based on a rose petal template PDMS substrate and surface deposited with a polypyrrole (PPy) active layer. Since the petal template has a millimeter/micro/nano structure, the device exhibits ultrahigh sensitivity (70 kPa^−1^) and stability in the detection range of 0.88 Pa–0.5 kPa. Guo et al. [31] successfully fabricated a high-aspect-ratio and low-density PDMS micro-tower array using a lotus leaf as the replication template. Then, ultrathin AgNWs were coated onto the micropatterned PDMS film as the electrode layer, and a flexible tactile sensor with high sensitivity (1.194 kPa^−1^) was obtained. Fang et al. [32] used PDMS to duplicate the microstructure of the lotus leaf surface and sprayed a graphene film on the surface to prepare a resistive tactile sensor with a large-area layered structure. The device showed a high sensitivity (1.2 kPa^−1^) over a wide linear range of 0.2–25 kPa. Table 1 lists the pressure sensing performances of sensors reported in the literature. However, these surface microstructures are unique and undesignable, so the flexible pressure sensors are inconsistent and cannot be modulated according to practical applications. Therefore, it is necessary to develop a method that can unrestrictedly change the shape, size, and spacing of the microstructures to make the obtained microstructures adjustable and accurate. Printed circuit boards (PCBs), widely used in various electronic products in daily life, are mainly composed of conductive copper foils and a substrate. A variety of surface morphology can be easily formed on a PCB surface that makes it suitable for making templates for microstructure fabrication.

In this paper, a simple and replicable fabrication method for flexible pressure sensors is proposed. Multiwalled carbon nanotubes (MWCNTs) with high conductivity and polyurethane (PU) with good tensile strength were used to prepare a microstructured composite nanomaterial film (MCNF) with regular batten microstructures via a mixed solution template method using a PCB template. Then, the MCNF was packaged with an interdigital electrode face-to-face to form a flexible pressure sensor. The dimensions of the batten microstructures were carefully tuned to study their effects on sensing performances. The experimental results show that the presented sensor is stable and highly sensitive in a wide measurement range, and it can detect both weak signal (wrist pulse) and large pressure (finger pressing and mouse clicking). Furthermore, the proposed method provides a simple, low-cost, and high-consistency technique for the fabrication of flexible pressure sensors.

## 2. Batten Microstructure Design and Simulation Analysis

Different structural parameters (width and spacing) of batten microstructures will lead to different contact areas with the interdigital electrodes, which makes the contact resistance different and affects the sensitivity and measurement range of the MCNF sensor. Therefore, the FEM analysis method was used to determine the relationship between the stress and microstrain of MCNFs with different dimensions under compressive loads, as well as the relationship between the contact area of the MCNF and the interdigital electrode and loads. These relationships can be used to illustrate the influence of different microstructure sizes on the sensitivity and measurement range of the MCNF sensor. Six models with different microstructures were designed, and the model parameters are shown in Table S1. Model-A, -B, and -C had microstructure heights of 70 μm, and Model-D, -E, -F, and -G had heights of 35 μm. Model-P is a planar structure film, which was added for comparison.

**Figure 1:**
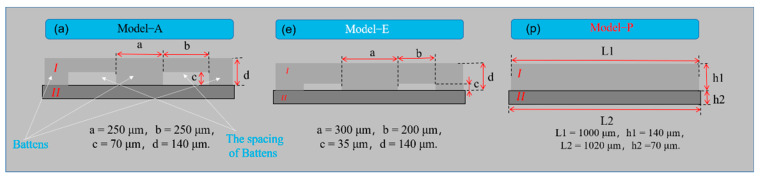
Schematic diagrams of the MCNFs contacting with the electrode (Model−A, Model−E, and Model−P).

**Figure 2:**
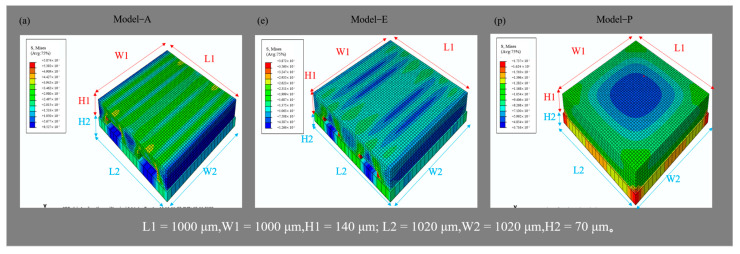
Stress distributions of MCNFs in contact with the electrodes of different models (Model−A, Model−E, and Model−P).

ABAQUS was used to model and analyze the different MCNF models and electrodes. The structure diagrams and dimensions of the models are shown in Figure 1 (only Model-A, -E, and -P are shown, and the diagrams of the other models are shown in Figure S1). In the established finite element model, *Ι* is the MCNF with a batten structure, with dimensions of 1 mm × 1 mm × 0.14 mm; *ΙΙ* is the polyimide (PI) electrode, which was simplified to a flat and solid film with dimensions of 1.02 mm × 1.02 mm × 0.07 mm. In Model-G, the dimensions of region *I* were 1.5 mm × 1.5 mm× 0.14 mm, and those of region *II* were 1.52 mm × 1.52 mm× 0.07 mm. Figure 2 shows the stress distributions of the different models (only Model-A, -E, -P, and results of other models are shown in Figure S2). During compression, multiple batten microstructures were gradually pressed on the surface of the electrode forming the interval contact structures, similar to bridges and piers. A stress concentration phenomenon appeared at the edge position, as shown in Figure 2 and Figure S2. This large local stress concentration increased the change of contact area during continuous compression, thereby improving the sensitivity of the sensor.

**Figure 3:**
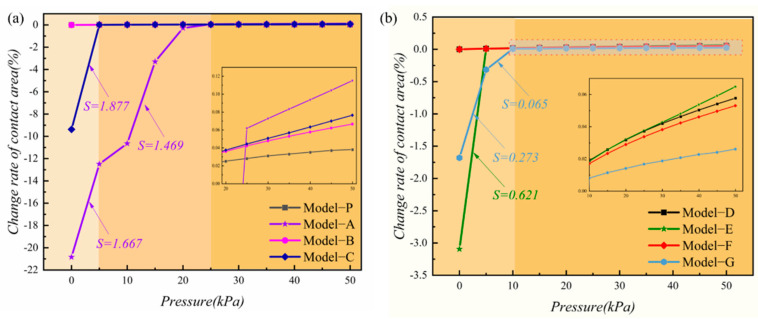
Change rate of contact area of different models under pressures from 0 to 50 kPa: (**a**) First set of models, Model−P and Models A−C; (**b**) The second group of models, Models D−G.

The stress–strain curves of different models were first obtained from the FEM analysis, as shown in Figure 3a. The results show that the models all exhibited linearity well in the stress range of 0–120 kPa, and the strains were close to each other due to the identical materials and similar batten microstructures of each model. Then, we focused on the strain rate–stress curves of each model to characterize the sensitivity of the strain responses of the models when they were subjected to compression. To facilitate the comparison, the models were divided into two groups based on the different heights of the battens, as shown in Figure 3b,c. The curves of the two groups show the same trend of a fast increase at the beginning and then a slow change. The strain rate with the microstructure model was significantly higher than that with no microstructure, indicating that the microstructured film is more prone to deformation. Furthermore, among the first set of models, the strain rate of Model-A was the largest. Because the line-width-to-spacing ratios of the batten microstructures of Model-A, -B, and -C were 1:1, 1.54:1, and 1.5:1, while other structural parameters were identical, we can infer that the smaller this ratio of the battens is, the larger the strain rate and response sensitivity are. A similar result was obtained in the second group, in which Model-G showed the largest strain rate and sensitivity because the width-to-spacing ratios of the batten microstructures of Model-D, -E, -F, and -G were 1.54:1, 1.5:1, 2:1, and 1:1, respectively.

When a pressure is applied on the sensor, it will lead to a deformation of the contact surfaces between the microstructured film and the electrode. This deformation will cause changes both in the contact area and the corresponding contact resistance of the film and electrode, which characterizes the sensor performance [33]. Therefore, the relationships between pressures and contact area change rates of the different models were studied for the MCNF’s optimal design. Figure 3a shows the variation in contact area under an increasing pressure for the models of the first group. The slope of the curves of contact area versus pressure can be used to characterize the sensitivity of involved sensors to some extent. Specifically, the slope of Model-A reached 1.667 in the pressure range of 0–5 kPa. Model-C had the largest slope in this range, with a value of 1.877. However, when the pressure exceeded 5 kPa, the change rate of the contact area of Model-C was no longer significant. However, Model-A continued to rise with a decreased slope of 1.469. As shown in the local enlarged diagram, when the pressure exceeded 25 kPa, the change rates of the contact areas of the four models from large to small were Model-A, -C, -B, and -P. However, the proportion of contact area for the four models were 0.5, 0.61, 0.6, and 1, respectively. This shows that the smaller the proportion of the contact area, the more concentrated the pressure in a certain range is and, therefore, the more significant the deformation of the MCNF. The deformation degree of the MCNF can directly affect the change of the contact resistance, thereby affecting the sensing performance.

The change in the contact area of models of the second group are shown in Figure 3b, and the proportion of the contact areas of Model-D, -E, -F, and -G were 0.61, 0.6, 0.7, and 0.97, respectively. The slope of the curve of Model-G was 0.273 within 0–5 kPa, and it decreased to 0.065 when the pressure increased to 10 kPa. In the local amplification diagram, the change rate of the contact area of Model-G was smaller than those of the other models when the pressure exceeded 5 kPa. This shows that, when the height of the batten structure is low, it cannot be easily deformed and can withstand high pressures. The contact area ratio of Model-E was the smallest, and the maximum slope of the curve at 0–5 kPa was 0.621. The second set of curves exhibited the same pattern as the first set, where the smaller the proportion of the contact area, the greater the corresponding sensitivity is. This may be attributed to the following possible reasons. When there is initial contact, an initial conductive pathway forms, with a corresponding resistance value. As the pressure increases, a sharp change in the contact area leads to more conductive pathways to reduce the resistance value, so the change in the contact area affects the sensor device sensitivity.

By performing the FEM analysis for the different models, it was shown that the dimensions of the microstructures affect the sensitivity of the sensor. First, the smaller the width-to-spacing ratio of the batten microstructure, the greater the strain rate of the corresponding MCNF flexible sensor is and the higher the response sensitivity becomes. Second, the larger the change rate of the contact area of the MCNF, the more easily it deforms and the more sensitive the piezoresistive response is. The results show that, for the first group of models, after the pressure exceeded 25 kPa, with a smaller proportion of contact area between the MCNF and electrode, the involved sensor exhibited a higher response sensitivity. The second group of models showed a negative correlation between the proportion of contact area and sensitivity after 5 kPa. From these results, we can see that Model-A, -E, and -G exhibited large sensitivities and large variation ranges. Therefore, Model-A, -D, -E, and -G were selected to fabricate flexible sensors for further study.

## 3. Experimental Section

### 3.1. Sensor Manufacturing

#### 3.1.1. Preparation of Microstructured Composite Nanomaterial Films (MCNFs)

**Figure 4:**
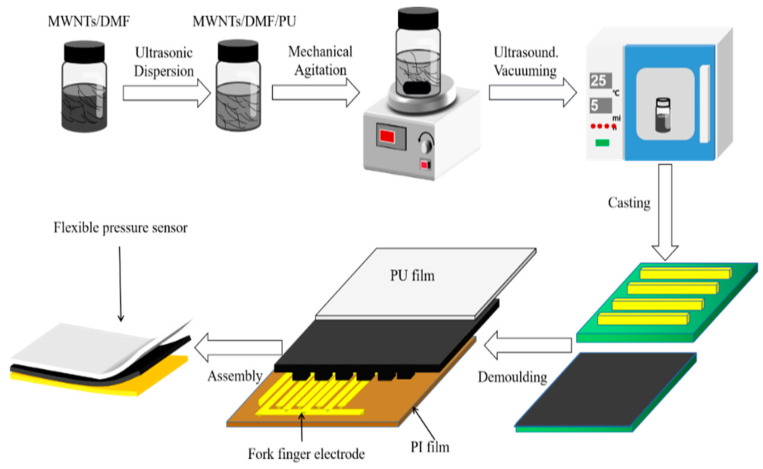
Preparation process of the MCNF flexible piezoresistive sensor.

**Figure 5:**
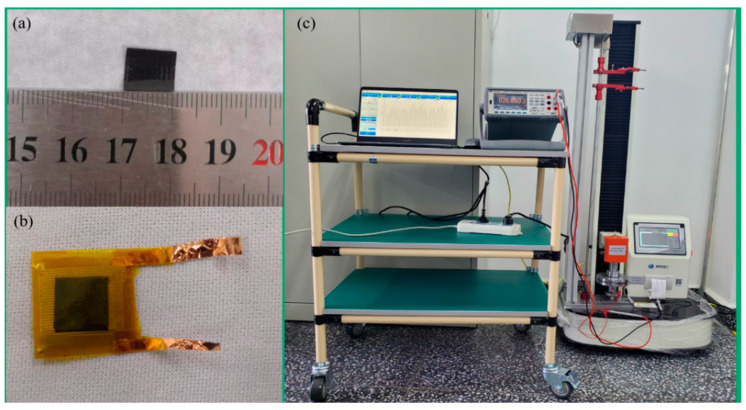
Pictures of the flexible sensor and experimental setup. (**a**) Size of the MCNF. (**b**) Optical image of a fabricated pressure sensor. (**c**) Measurement system for the flexible pressure sensor.

MCNFs were prepared using a mixed solution template method. The preparation process is shown in Figure 4. First, a certain amount of multiwalled carbon nanotubes (MWCNTs, external diameters: 30–80 nm, lengths: >10 μm, and purity > 98%, purchased from Chengdu Organic Chemistry Co., Ltd., Chengdu, China) were dispersed to 5 mL of dimethylformamide (DMF, purchased from Chinasun Specialty Products Co.,Ltd., Changshu, China) by ultrasonic dispersion for 1 h. Then, the suspension was dropped into 9 g of waterborne polyurethane (PU, with a solid content of 32 ± 5% and viscosity < 300 mPa·s, purchased from Shenzhen Jitian Chemical Co., Ltd., Shenzhen, China). The mixture was mechanically stirred for 4 h and then ultrasonically dispersed for another 1 h. After the completion of three vacuum extractions of bubbles, each lasting 3–5 min, the mixture was finally evenly coated on the PCB template. After curing at room temperature, the composite films were peeled off, and MCNFs with microstructures were obtained. The dimensions of the MCNF films were 10 mm × 10 mm, as shown in Figure 5a.

#### 3.1.2. Preparation of Single-Sided Interdigital Electrode

The interdigital electrode was fabricated using flexible printed circuit board technology, and polyimide (PI) was used as the flexible substrate for its high strength and flexibility. To conveniently read the change in resistance, two conductive copper foils were glued at both ends of the electrode with conductive silver adhesive. Then, a heat-resistant tape (Kailepai) was used to ensure the adhesion and reinforce the connection.

#### 3.1.3. Fabrication of Pressure Sensor

A piece of MCNF and a flexible interdigital electrode were placed together face-to-face, and another pure PU film was then formed on the surface of the active layer to prevent it from deterioration failure. After that, the sensor was packaged using a Kailepai tape. The assembled flexible pressure sensor is shown in Figure 5b, and the thickness of the entire sensor was about 0.6 mm.

### 3.2. Characterization and Measurement

Due to the influence of the surface microstructure of the sensor on its performance, the microstructure was examined by scanning electron microscopy (SEM, GeminiSEM500, Carl Zeiss Microscopy GmbH, Jena, Germany). A measurement system was constructed to test the pressure-response of the fabricated flexible sensor, as shown in Figure 5c, which mainly included a universal testing machine (ZQ−990/LB, Zhiqu Precision Instruments Co., Ltd., Dongguan, China), a digital multimeter (Keysight 34465A, Keysight Technologies, Inc., Santa Rosa, CA, USA), a computer, and the control software. Before the measurement started, a 15 mm × 15 mm × 1.1 mm glass cover plate was first placed over the sensor, and the corresponding initial pressure was 59.9 Pa. The glass cover plate could make the applied pressure more even. To characterize the piezoresistive performance of the fabricated flexible pressure sensor, an increasing pressure was loaded on it, and the resistance variation of the sensor was recorded at the same time. After that, a cyclic loading test was used to investigate the stability of the sensor.

## 4. Results and Discussions

### 4.1. Sensing Mechanism Analysis

**Figure 6:**
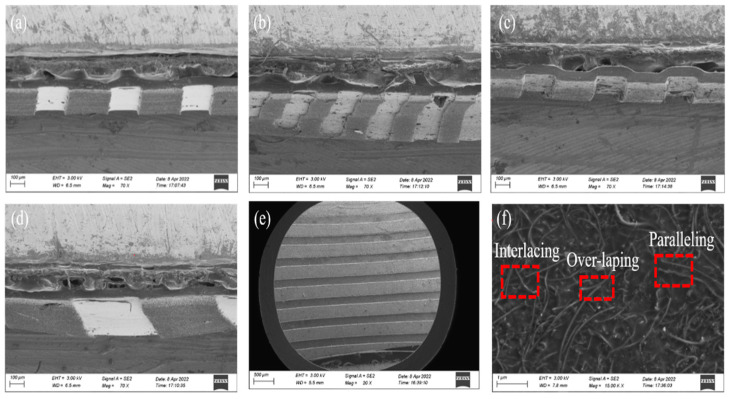
Scanning electron microscopy (SEM) images of MCNFs. Cross-sectional microstructures of (**a**) Model−A, (**b**) Model−D, (**c**) Model−E, and (**d**) Model−G. (**e**) SEM image of the bottom surface of a Model−G MCNF. (**f**) SEM image of the MWCNTs on the bottom surface of an MCNF.

MCNFs with the microstructures of Model-A, -D, -E, and -G described in Section 2 were fabricated, respectively. Figure 6a–d show the SEM images of the MCNF cross-sections. It is clear that the structures of the prepared MCNFs were consistent with the templates, indicating the feasibility of using the PCB as a template to mold batten microstructures on MCNFs. Figure 6e shows the bottom surface of the MCNF, from which it can be seen that the widths and spacing of the battens were regular and consistent, and the microstructures were uniformly distributed. Figure 6f shows the SEM image of the distribution of MWCNTs on the bottom surface of the MCNF, on which the MWCNTs were randomly distributed. A variety of position relationships among the MWCNTs, such as overlapping, paralleling, and interlacing, can be observed in the image.

**Figure 7:**
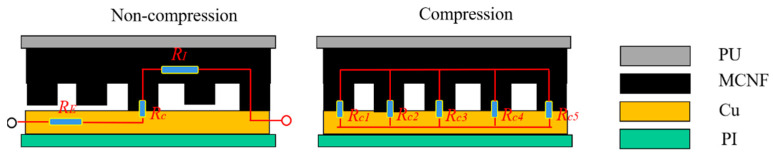
Response mechanism of MCNF pressure sensors.

The response mechanism of the MCNF pressure sensor generally includes two different cases: non-compression and compression, as shown in Figure 7. Under non-compression, the contact resistance (*R_C_*) between the MCNF and electrodes is much larger than the resistance (*R_I_*) inside the MCNF itself [18]. When a small pressure is applied, the contact resistance between the MCNF and electrode is significantly reduced, which was a key factor affecting the initial sensitivity of the sensor. The initial total resistance of the pressure sensor can be expressed as:

*R_T_* = *R_I_* + *R_C_* + *R_E_* (1)

where *R_T_* is the total resistance, *R_I_* is the internal resistance of the MCNF, *R_C_* is the contact resistance between the MCNF and the electrode, and *R_E_* is the resistance of the copper electrode. When a pressure is applied, the change of the *R_C_* value, Δ*R_C_*, can be calculated as follows:

Δ*R*’*_C_* = *R_C1_*//*R_C2_*//*R_C3_*//*R_C4_*//……//*R_Cn_* (2)

Under high pressures, the change of the contact area between the MCNF and electrode tends to be stable, and the contact resistance approaches to a constant. In this situation, the piezoresistivity of the MCNF becomes the dominant factor governing the performance of the sensor.

### 4.2. Performance Testing of the Pressure Sensor

The contents of the MWCNTs in MCNFs determines the piezoresistivity of the MCNFs. Four different samples were prepared with different weight ratios of MWCNTs and PU. The corresponding samples were named MWCNT_0.022_/PU, MWCNT_0.032_/PU, MWCNT_0.042_/PU, and MWCNT_0.052_/PU, respectively. The specific component contents are listed in Table S2.

**Figure 8:**
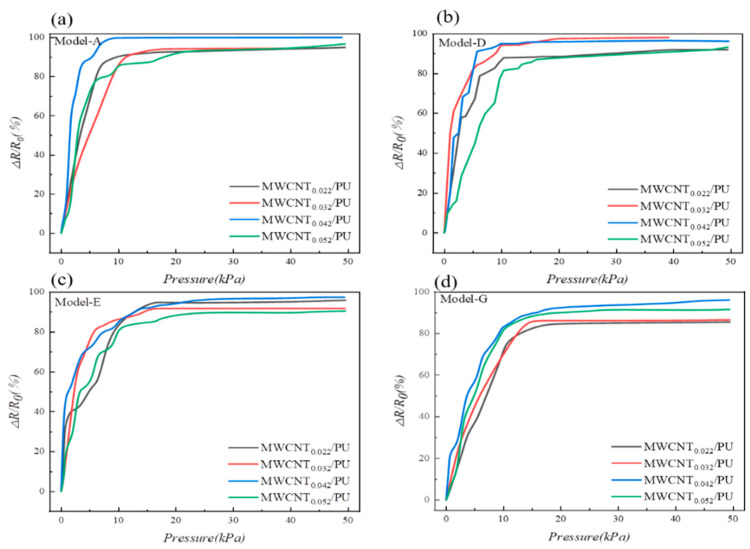
Effect of MWCNT content on pressure-response of different models. (**a**) Model−A, (**b**) Model−D, (**c**) Model−E, and (**d**) Model−G.

To analyze the effect of MWCNT content in the MCNFs on the sensors’ sensitivity, the pressure–response curves of four models with different filler contents were compared. From the pressure−response curves shown in Figure 8, we can find that all the curves can be divided into two sections: an initial ascending region and a following flat region. All the samples showed an increasing sensitivity as the MWCNT content increased from 0.022 to 0.042. The MWCNT_0.042_/PU exhibited the highest sensitivity at lower pressures (<5 kPa) except for Model-D. This indicates that, for the same microstructure of the MCNF, the higher the MWCNT content, the greater the rate of change of the piezoresistive resistance is during compression. This results in a significant increase in sensitivity during the application of pressure. However, as the MWCNT content increased to 0.052, the sensitivity did not continue to increase but tended to decrease. This indicates that, if the MWCNT content is too high, more carbon nanotubes overlap with each other; then, the conductive paths are stabler, and the resistance changes less under pressure. Therefore, MWCNT_0.042_/PU was chosen for the fabrication of subsequent sensors.

**Figure 9:**
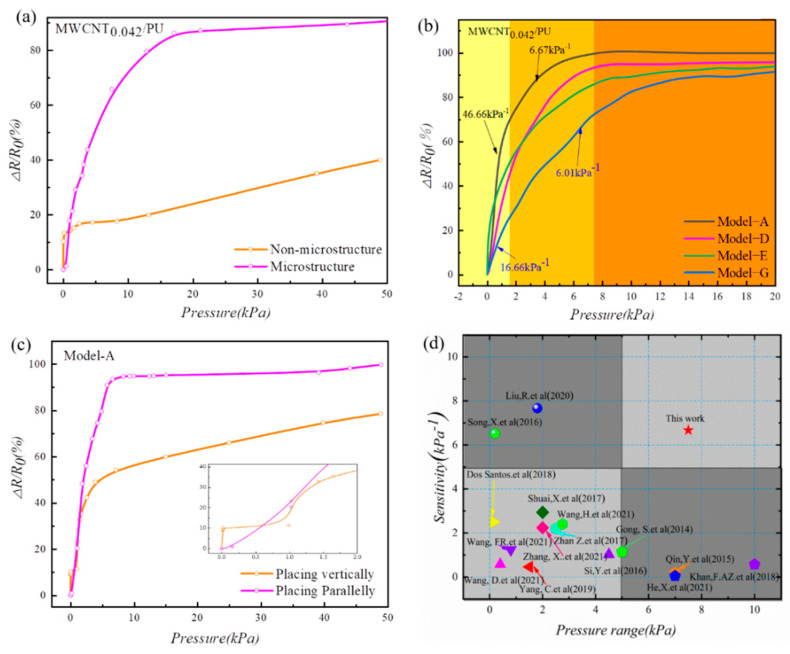
Sensing performance of the presented sensors. (**a**) Pressure-response of the sensor with and without microstructures. (**b**) Pressure-responses of MCNF sensors with the same MWCNT contents and different microstructures. (**c**) Pressure-response of MCNF sensor placed vertically and parallel to electrodes. (**d**) Comparison of the sensitivities and operating ranges in the literature and our work.

The effectiveness of improving sensitivity by introducing microstructures into the surfaces of MCNFs was proven by the FEM analysis presented in Section 2. In order to illustrate the impact of the designed microstructure on the sensing performance of the flexible pressure sensor, the pressure-response was compared with a non-microstructured sensor. Figure 9a shows the pressure and resistance variation relationships for the sensors with planar and microstructured MCNFs. Both films were MWCNT_0.042_/PU, and the microstructure was based on Model-A. In the range of 0–0.9 kPa, the sensitivity of the planar sensor was higher than that of the microstructured one. This may be attributed to the contact part of the former with the interdigital electrode being much larger than that of the latter, resulting in a far smaller initial resistance value than that of the latter. Then, when a small pressure was applied, the formation of conductive paths between the two contact surfaces was greatly promoted, so the variation in contact resistance of the planar sensor was greater than that of the microstructured sensor. However, with the increase in pressure, the pressure–resistance curve of the planar structure slid into a state of slow change. On the other side, the MCNF sensor with the batten microstructures gradually deformed and continuously formed complete contact with the electrode as pressure continuously increased. When the pressure continued to increase, each batten microstructure plastically extended to both sides at the contact position, thus further increasing the contact area, causing the response curve to continue to grow.

The sensitivities of the MCNF sensors with different microstructures with the same MWCNT content were also different. Therefore, to verify the effect of the microstructure on the sensor’s sensitivity, the MWCNT_0.042_/PU MCNF was selected to compare the pressure-response of the sensor with different microstructures, as shown in Figure 9b. The pressure-response of the sensor based on Model-A showed a high sensitivity of 46.66 kPa^−1^ in the range of 0–1.5 kPa, followed by a nonlinear region with a sensitivity of 6.67 kPa^−1^ above 1.5 kPa. In addition, the sensitivity remained almost constant when pressure was greater than 7.5 kPa. In the pressure range of 0–1.5 kPa, the sensor based on Model-G exhibited the minimum sensitivity of 16.67 kPa^−1^. In addition, it still presented a sensitivity of 6.01 kPa^−1^ in the pressure range of 1.5–12 kPa, demonstrating its high sensitivity over a large range. Consequently, sensors fabricated based on the Model-A were selected for the subsequent investigations.

Both the MCNF and the interdigital electrodes have one-dimensional microstructures on the contact surfaces. In order to analyze the impact of the relative positions of the two one-dimensional microstructures on the sensitivity of the flexible sensor, a comparative experiment was performed. Figure 9c shows the pressure–response curve when the MCNF was placed perpendicularly or parallelly to the interdigital electrode. When the pressure was below 0.5 kPa, the response curve of the MCNF placed perpendicularly increased rapidly, showing a higher sensitivity. The perpendicular placement formed a cross-vertical staggered network structure in the process of compression, and the resistance changed significantly. However, the effective conductive path decreased with the change of the contact area during the gradual increase in pressure, and the response curve tended to be flat after 3 kPa. The pressure–response curve of the MCNF with a parallel placement showing a good linearity in the range of 0–9 kPa indicates that parallel placement can effectively increase the linear measurement range. Therefore, parallel placement was used in the subsequent process. Figure 9d compares the sensing performance of the pressure sensor presented in this paper with those previously reported in the literature [28,34,35,36,37,38,39,40,41,42,43,44,45,46,47]. The device designed in this paper achieved an optimized pressure sensing performance with a high sensitivity and a wide measurement range. This can be attributed to its unique batten microstructures and high-performance MCNFs.

**Figure 10:**
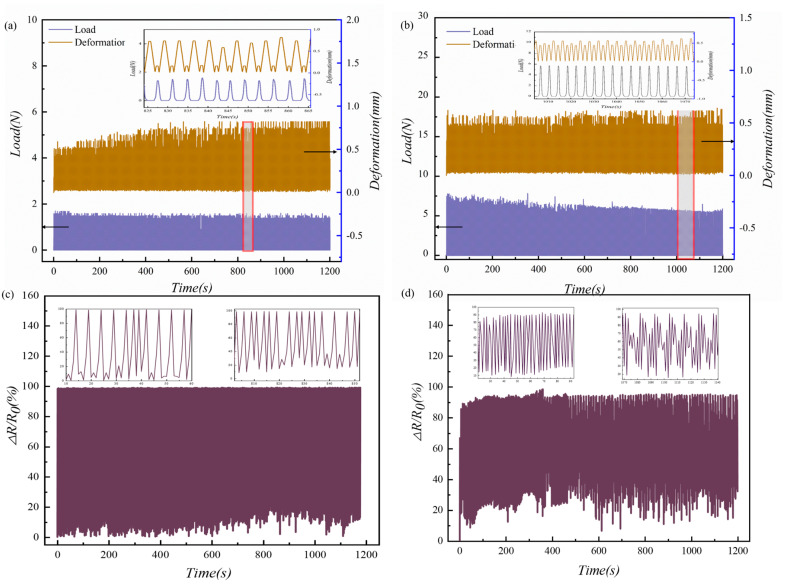
Cyclic loading experiment results of the presented flexible pressure sensors: Model−A cyclically loaded with (**a**) 0–1 N and (**b**) 0–5 N; piezoresistive response curves of (**c**) Model−A and (**d**) Model-E at 0–10 kPa under cyclic loading.

Cyclic loading experiments were performed to investigate the stability of the fabricated sensor. Figure 10a,b shows the load and deformation versus time curves for the MCNF sensors of Model-A. Two different loads, 0–10 kPa and 0–50 kPa, respectively, were employed to actuate the sensor individually. Every experiment comprised 500 loading–unloading cycles experienced about 20 min. The resistance values were collected under cyclic loading at a frequency of 10 Hz. In Figure 10c,d, the resistance variation versus pressure curves of Model-A and Model-E under 0–10 kPa cyclic loading are shown, respectively. The sensing performances of both models remained highly stable over multiple cycles. More specifically, the sensor based on Model-A demonstrated superiority in terms of stability. These results verify that the MCNF flexible sensor has a high mechanical stability and long-term durability. The reliability of the sensor can be attributed to the structural stability of the batten microstructures, the mechanical flexibility of the PU substrate, and the robustness of the packaging.

### 4.3. Practical Application of the Pressure Sensor

**Figure 11:**
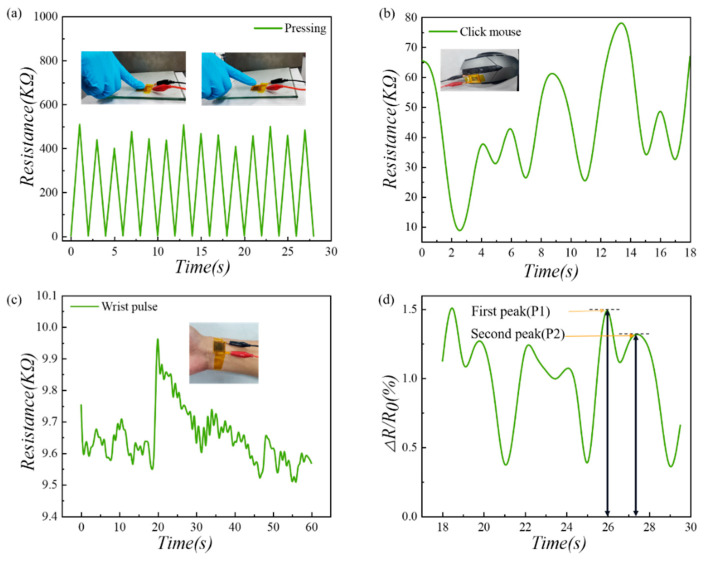
Applications of the batten microstructure pressure sensor in real-time monitoring of human activities: (**a**) Fingers pressing; (**b**) Mouse clicking; (**c**) Real-time pulse detecting; (**d**) Pulse vibration waveform.

Thanks to the good performances of the fabricated sensor, it could be used to detect pressure variations induced by different human activities. Sensors based on Model-A microstructures were employed in this series of experiments. First, a sensor was fixed on a glass cover plate, and a finger alternately pressed and released the surface of the sensor. As shown in Figure 11a, when the finger pressed, the resistance dropped sharply to the minimum value; when the finger moved away, the resistance quickly recovered to the maximum value. The stability of the sensor was also demonstrated in the entire process. Then, we stuck a sensor to the left button of a mouse and clicked it continuously and randomly. As shown in Figure 11b, both the click speed and compression strength could be recorded simultaneously by the sensor.

Since the sensor possessed excellent linearity and sensitivity in the low and medium pressure ranges, it could be used for the real-time detection of the pulse signal from the wrist artery. A 24-year-old male, 174 cm in height and 64 kg in weight, was selected as a volunteer in this experiment (the volunteer involved in the collection of human physiological data was informed and agreed). As shown in Figure 11c, the sensor was fixed on the inner wrist of the volunteer with tape. In the test, the volunteer performed a stretching movement of the hand, which corresponded to a large, abrupt change in the resistance value around the 20th second in the figure. As the volunteer’s hand returned to a neutral position, the resistance decreased to the initial value. An intercepted portion of the curve of the resistance change is shown in Figure 11d. From this figure, the first and second peak values corresponding to the peak pressure of the wrist pulse in the early and late systolic phase are clearly indicated. This allows the derivation of the radial augmentation index (*AIr*), a parameter used for health monitoring, which is a characteristic value of arterial stiffness and is closely related to the age of the person [48]. In the waveform, the first peak is denoted as *P*_1_, and the second peak is denoted as *P*_2_. The ratio *AIr* = *P*_2_/*P*_1_ = 0.7, which is a youthful health characteristic value.

## 5. Conclusions

In this paper, we presented an ultrasensitive flexible pressure sensor based on MCNF with batten microstructures through a facile method. The adopted batten microstructures provide a reduced modulus of elasticity and microscale roughness at the same time. It therefore significantly increases the contact area between the MCNFs and the interdigital electrodes as pressure is applied to it. Due to the advantages of the structure design and accurate template, the designed sensor exhibits significantly high sensitivities of 46.66 kPa^−1^ from 0 to 1.5 kPa and 6.67 kPa^–1^ from 1.5 to 7.5 kPa. Furthermore, excellent performances, including high stability and flexibility, were experimentally verified. Additionally, we demonstrated the usability of the sensor in several applications for physiological signal monitoring of tactile pressure measurements. With the facile and low-cost fabrication method, the flexible pressure sensor may be suitable for various applications, including human–machine interfaces and wearable devices.

## Figures and Tables

**Table 1:** Pressure sensing performances of sensors reported in the literature.

Sensing Materials	Operation Type	Sensitivity (kPa^−1^)	Working Range (kPa)	Ref
Silver/PDMS	Resistive	3.2	<1	[23]
MWNTs/PDMS	Resistive	29	>10	[24]
CNT/CB/Silicone Rubber	Resistive	1.2	0–1800	[25]
AgNWs/PDMS	Resistive	55	0–100	[27]
		>10	100–400	
Carbon Coating/Silver/PDMS	Resistive	2.5	0–0.16	[28]
Graphene/PDMS	Resistive	5.53	0–1.4	[29]
PPy/PDMS	Resistive	120 (<0.5 kPa)	0.88–32	[30]
AgNWs/PDMS	Capacitive	1.194	0–2	[31]
		0.077	2–14	
Graphene/PDMS	Resistive	1.25	0–25	[32]

## Data Availability

All data needed to evaluate the conclusions in the paper are present in the paper and/or the Supplementary Materials.

## Supplementary Materials

The following supporting information can be downloaded at: https://www.mdpi.com/article/10.3390/mi13081164/s1. Table S1: dimensions of the models; Table S2: compositions of MCNFs; Figure S1: schematic diagrams of the MCNF in contact with the electrode title; Figure S2: stress distributions of MCNFs in contact with electrodes of different models; Figure S3: FEM analysis results of MCNFs.

## References

[1] Wang C., Hwang D., Yu Z., Takei K., Park J., Chen T., Ma B., Javey A. (2013). User-Interactive Electronic Skin for Instantaneous Pressure Visualization. Nat. Mater..

[2] Mannsfeld S.C.B., Tee B.C.-K., Stoltenberg R.M., Chen C.V.H.-H., Barman S., Muir B.V.O., Sokolov A.N., Reese C., Bao Z. (2010). Highly Sensitive Flexible Pressure Sensors with Microstructured Rubber Dielectric Layers. Nat. Mater..

[3] Tee B.C.-K., Chortos A., Berndt A., Nguyen A.K., Tom A., McGuire A., Lin Z.C., Tien K., Bae W.-G., Wang H. (2015). A Skin-Inspired Organic Digital Mechanoreceptor. Science.

[4] Kim D.-H., Lu N., Ma R., Kim Y.-S., Kim R.-H., Wang S., Wu J., Won S.M., Tao H., Islam A. (2011). Epidermal Electronics. Science.

[5] Wang X., Dong L., Zhang H., Yu R., Pan C., Wang Z.L. (2015). Recent Progress in Electronic Skin. Adv. Sci..

[6] Tai L.C., Gao W., Chao M.H., Bariya M., Ngo Q.P., Shahpar Z., Nyein H.Y.Y., Park H., Sun J., Jung Y. (2018). Methylxanthine drug monitoring with wearable sweat sensors. Adv. Mater..

[7] Roy A.L. (2017). Innovation or violation? Leveraging mobile technology to conduct socially responsible community research. Am. J. Commun. Psychol..

[8] Chen M., Ma Y., Song J., Lai C.F., Hu B. (2016). Smart clothing: Connecting human with clouds and big data for sustainable health monitoring. Mobile Netw. Appl..

[9] Santos G.L., Endo P.T., Tigre M.F.F.D.L., da Silva L.G.F., Sadok D., Kelner J., Lynn T. (2018). Analyzing the availability and performance of an e-health system integrated with edge, fog and cloud infrastructures. J. Cloud Comput..

[10] Cha Y., Seo J., Kim J.S., Park J.M. (2017). Human–computer interface glove using flexible piezoelectric sensors. Smart Mater. Struct..

[11] Hsu F.R., Kuo Y.H., Wei S.Y., Hsieh Y.H., Nguyen D.C. (2019). A study of user interface with wearable devices based on computer vision. IEEE Consum. Electron. Mag..

[12] Huo X., Park H., Kim J., Ghovanloo M. (2013). A dual-mode human computer interface combining speech and tongue motion for people with severe disabilities. IEEE Trans. Neural Syst. Rehabil. Eng..

[13] Ma Z., Li S., Wang H.T., Cheng W., Li Y., Pan L.J., Shi Y. (2019). Advanced electronic skin devices for healthcare applications. J. Mater. Chem. B.

[14] Park J., Lee Y., Ha M., Cho S., Ko H. (2016). Micro/nanostructured surfaces for self-powered and multifunctional electronic skins. J. Mater. Chem. B.

[15] Yin Y.F., Cui Y., Li Y.H., Xing Y.F., Li M. (2018). Thermal management of flexible wearable electronic devices integrated with human skin considering clothing effect. Appl. Therm. Eng..

[16] Luo S., Zhou X., Tang X.Y., Li J.L., Wei D.C., Tai G.J., Chen Z.Y., Liao T.M., Fu J.T., Wei D.P. (2021). Microconformal electrode-dielectric integration for flexible ultrasensitive robotic tactile sensing. Nano Energy.

[17] Zhu B., Ling Y., Yap L.W., Yang M., Lin F., Gong S., Wang Y., An T., Zhao Y., Cheng W. (2019). Hierarchically Structured Vertical Gold Nanowire Array-Based Wearable Pressure Sensors for Wireless Health Monitoring. ACS Appl. Mater. Interfaces.

[18] Choong C.L., Shim M.B., Lee B.S., Jeon S., Ko D.S., Kang T.H., Bae J., Lee S.H., Byun K.E., Im J. (2014). Highly stretchable resistive pressure sensors using a conductive elastomeric composite on a micropyramid array. Adv. Mater..

[19] Luo S., Yang J., Song X., Zhou X., Yu L., Sun T., Yu C., Huang D., Du C., Wei D. (2018). Tunable-sensitivity flexible pressure sensor based on graphene transparent electrode. Solid State Electron..

[20] Cheng W., Wang J., Ma Z., Yan K., Wang Y., Wang H., Li S., Li Y., Pan L., Shi Y. (2017). Flexible pressure sensor with high sensitivity and low hysteresis based on a hierarchically microstructured electrode. IEEE Electron Device Lett..

[21] Ji B., Zhou Q., Wu J., Gao Y., Wen W., Zhou B. (2020). Synergistic Optimization toward the Sensitivity and Linearity of Flexible Pressure Sensor via Double Conductive Layer and Porous Microdome Array. ACS Appl. Mater. Interfaces.

[22] Dos Santos A., Pinela N., Alves P., Santos R., Farinha R., Fortunato E., Martins R., Águas H., Igreja R. (2019). E-skin bimodal sensors for robotics and prosthesis using PDMS molds engraved by laser. Sensors.

[23] Sun X.G., Sun J.H., Zheng S.K., Wang C.K., Tan W.S., Zhang J.G., Liu C.X., Liu C., Li T., Qi Z.M. (2019). A sensitive piezoresistive tactile sensor combining two microstructures. Nanomaterials.

[24] Park J., Kim J., Hong J., Lee H., Lee Y., Cho S., Kim S.W., Kim J.J., Kim S.Y., Ko H. (2018). Tailoring force sensitivity and selectivity by microstructure engineering of multidirectional electronic skins. NPG Asia Mater..

[25] Huang Y., Wang W.H., Sun Z.G., Wang Y., Liu P., Liu C.X. (2015). A multilayered flexible piezoresistive sensor for wide-ranged pressure measurement based on CNTs/CB/SR composite. J. Mater. Res..

[26] Chen X., Shao J., Tian H., Li X., Wang C., Luo Y., Li S. (2020). Scalable Imprinting of Flexible Multiplexed Sensor Arrays with Distributed Piezoelectricity-Enhanced Micropillars for Dynamic Tactile Sensing. Adv. Mater. Technol..

[27] Zhang T., Li Z., Li K., Yang X. (2019). Flexible pressure sensors with wide linearity range and high sensitivity based on selective laser sintering 3D printing. Adv. Mater. Technol..

[28] dos Santos A., Pinela N., Alves P., Santos R., Fortunato E., Martins R., Aguas H., Igreja R. (2018). Piezoresistive E-skin sensors produced with laser engraved molds. Adv. Electron. Mater..

[29] Zhu B.W., Niu Z.Q., Wang H., Leow W.R., Wang H., Li Y.G., Zheng L.Y., Wei J., Huo F.W., Chen X.D. (2014). Microstructured graphene arrays for highly sensitive flexible tactile sensors. Small.

[30] Yu S.X., Li L.L., Wang J.J., Liu E.P., Zhao J.X., Xu F., Cao Y.P., Lu C.H. (2020). Light-Boosting Highly Sensitive Pressure Sensors Based on Bioinspired Multiscale Surface Structures. Adv. Funct. Mater..

[31] Wan Y.B., Qiu Z.G., Hong Y., Wang Y., Zhang J.M., Liu Q.X., Wu Z.G., Guo C.F. (2018). A highly sensitive flexible capacitive tactile sensor with sparse and high-aspect-ratio microstructures. Adv. Electron. Mater..

[32] Shi J.D., Wang L., Dai Z.H., Zhao L.Y., Du M.D., Li H.B., Fang Y. (2018). Multiscale hierarchical design of a flexible piezoresistive pressure sensor with high sensitivity and wide linearity range. Small.

[33] Wang X., Gu Y., Xiong Z., Cui Z., Zhang T. (2014). Silk-molded flexible, ultrasensitive, and highly stable electronic skin for monitoring human physiological signals. Adv. Mater..

[34] Zhan Z.Y., Lin R.Z., Tran V.T., An J.N., Wei Y.F., Du H.J., Tran T., Lu W. (2017). Paper/carbon nanotube-based wearable pressure sensor for physiological signal acquisition and soft robotic skin. ACS Appl. Mater. Interfaces.

[35] Khan F.A., Ajmal C.M., Bae S., Seo S., Moon H., Baik S. (2018). Silver nanoflower decorated graphene oxide sponges for highly sensitive variable stiffness stress sensors. Small.

[36] Qin Y.Y., Peng Q.Y., Ding Y.J., Lin Z.S., Wang C.H., Li Y., Li J.J., Yuan Y., He X.D., Li Y.B. (2015). Lightweight, superelastic, and mechanically flexible graphene/polyimide nanocomposite foam for strain sensor application. ACS Nano.

[37] Si Y., Wang X.Q., Yan C.C., Yang L., Yu J.Y., Ding B. (2016). Ultralight biomass-derived carbonaceous nanofibrous aerogels with superelasticity and high pressure-sensitivity. Adv. Mater..

[38] He X., He X., He H.L., Liang S.L., Liu Z.H., Liang J.H., Xin Y., Yang W.J., Chen Y., Zhang C. (2021). Large-Scale, Cuttable, Full Tissue-Based Capacitive Pressure Sensor for the Detection of Human Physiological Signals and Pressure Distribution. ACS Omega.

[39] Liu R., Li J.M., Li M., Zhang Q.H., Shi G.Y., Li Y.G., Hou C.Y., Wang H.Z. (2020). MXene-coated air-permeable pressure-sensing fabric for smart wear. ACS Appl. Mater. Interfaces.

[40] Wang F., Tan Y., Peng H., Meng F., Yao X. (2021). Investigations on the preparation and properties of high-sensitive BaTiO3/MwCNTs/PDMS flexible capacitive pressure sensor. Mater. Lett..

[41] Yang C.X., Liu W.J., Liu N.S., Su J., Li L.Y., Xiong L., Long F., Zou Z.G., Gao Y.H. (2019). Graphene aerogel broken to fragments for a piezoresistive pressure sensor with a higher sensitivity. ACS Appl. Mater. Interfaces.

[42] Zhang X., Chen F., Han L., Zhang G., Hu Y., Jiang W., Zhu P., Sun R., Wong C.P. (2021). Flexible, highly sensitive, and ultrafast responsive pressure sensor with stochastic microstructures for human health monitoring. Adv. Eng. Mater..

[43] Shuai X., Zhu P., Zeng W., Hu Y., Liang X., Zhang Y., Sun R., Wong C.P. (2017). Highly sensitive flexible pressure sensor based on silver nanowires-embedded polydimethylsiloxane electrode with microarray structure. ACS Appl. Mater. Interfaces.

[44] Song X.F., Sun T., Yang J., Yu L.Y., Wei D.C., Fang L., Lu B., Du C.L., Wei D.P. (2016). Direct growth of graphene films on 3D grating structural quartz substrates for high-performance pressure-sensitive sensors. ACS Appl. Mater. Interfaces.

[45] Gong S., Schwalb W., Wang Y.W., Chen Y., Tang Y., Si J., Shirinzadeh B., Cheng W.L. (2014). A wearable and highly sensitive pressure sensor with ultrathin gold nanowires. Nat. Commun..

[46] Wang H., Cen Y., Zeng X. (2021). Highly Sensitive Flexible Tactile Sensor Mimicking the Microstructure Perception Behavior of Human Skin. ACS Appl. Mater. Interfaces.

[47] Wang D., Zhou X., Song R.F., Fang C.Q., Wang Z., Wang C.X., Huang Y.W. (2021). Freestanding silver/polypyrrole composite film for multifunctional sensor with biomimetic micropattern for physiological signals monitoring. Chem. Eng. J..

[48] Nichols W.W. (2005). Clinical measurement of arterial stiffness obtained from noninvasive pressure waveforms. Am. J. Hypertens..

