# Supplementary material for: Facile Fabrication of a Highly Sensitive and Robust Flexible Pressure Sensor with Batten Microstructures
Source: Micromachines (Basel). 2022 Jul 23;13(8):1164. doi: 10.3390/mi13081164 (PMC9329788; doi:10.3390/mi13081164)
Supplement: Supplementary file 1 [file micromachines-13-01164-s001.zip › micromachines-1821453-supplementary.pdf]

Supplementary information

Table S1. Dimensions of the models.

Model	Line width of batten (μm)	Spacing of batten (μm)	Height of batten (μm)
model-A	250	250	70
model-B	200	130	70
model-C	300	200	70
model-D	200	130	35
model-E	300	200	35
model-F	200	100	35
model-G	500	500	35
model-P	-	-	-

Table S2. Compositions of MCNFs.

Sample name	PU (g)	MWCNTs (g)
$MWCNT_{0.022}$/PU	0.9	0.02
$MWCNT_{0.032}$/PU	0.9	0.03
$MWCNT_{0.042}$/PU	0.9	0.04
$MWCNT_{0.052}$/PU	0.9	0.05

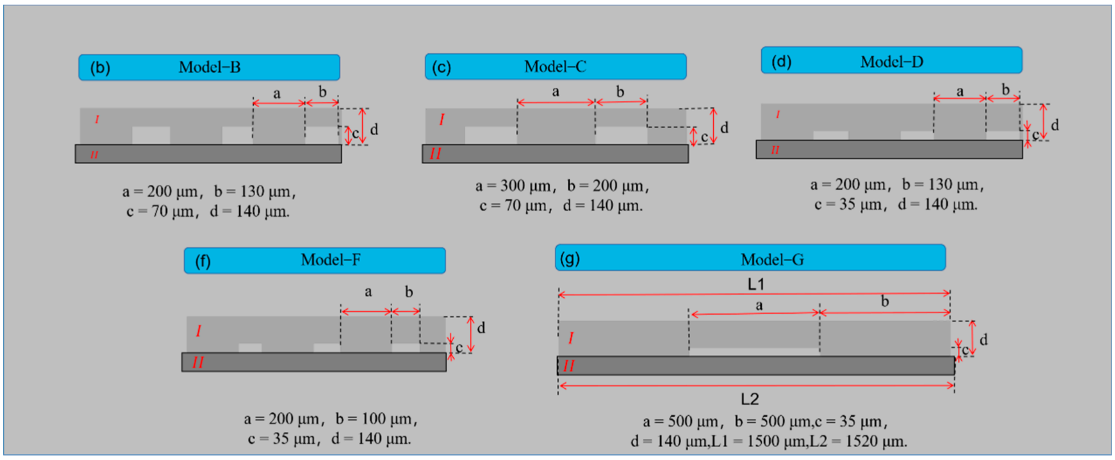

Figure S1. Schematic diagrams of the MCNF in contact with the electrode (Model-B, Model-C, Model-D, Model-F, and Model-G).

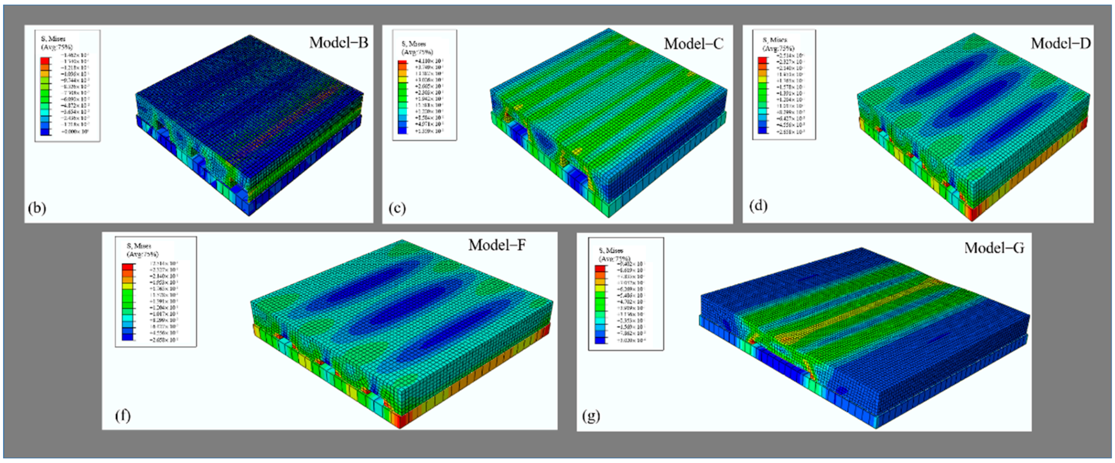

Figure S2. Stress distributions of MCNFs in contact with electrodes of different models (Model-B, -C, -D, -F, and -G).

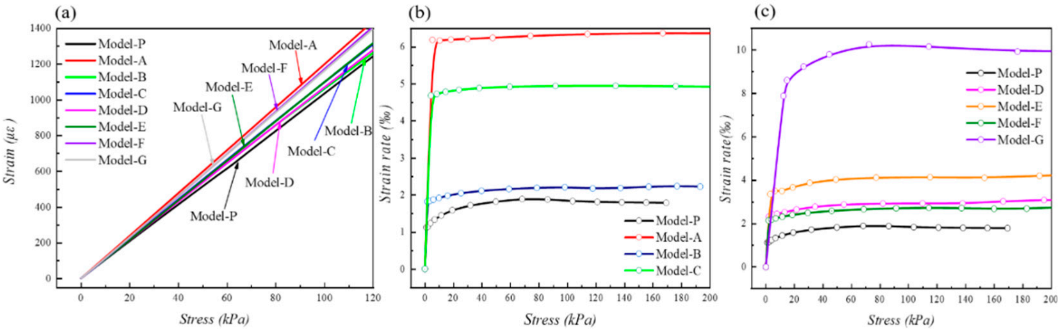

Figure S3. FEM analysis results of MCNFs. (a) Stress–micro-strain curves of finite element Model-P and Model-A through Model-G; (b) Stress–strain rate curves of finite element Model-P and Model-A through Model-C; (c) Stress–strain rate curves of finite element Model-P and Model-D through Model-G.
